# Supplementary figures and images for: Reduced IFN-ß inhibitory activity of Lagos bat virus phosphoproteins in human compared to Eidolon helvum bat cells
Source: PLoS One. 2022 Mar 8;17(3):e0264450. doi: 10.1371/journal.pone.0264450 (PMC8903296; doi:10.1371/journal.pone.0264450)

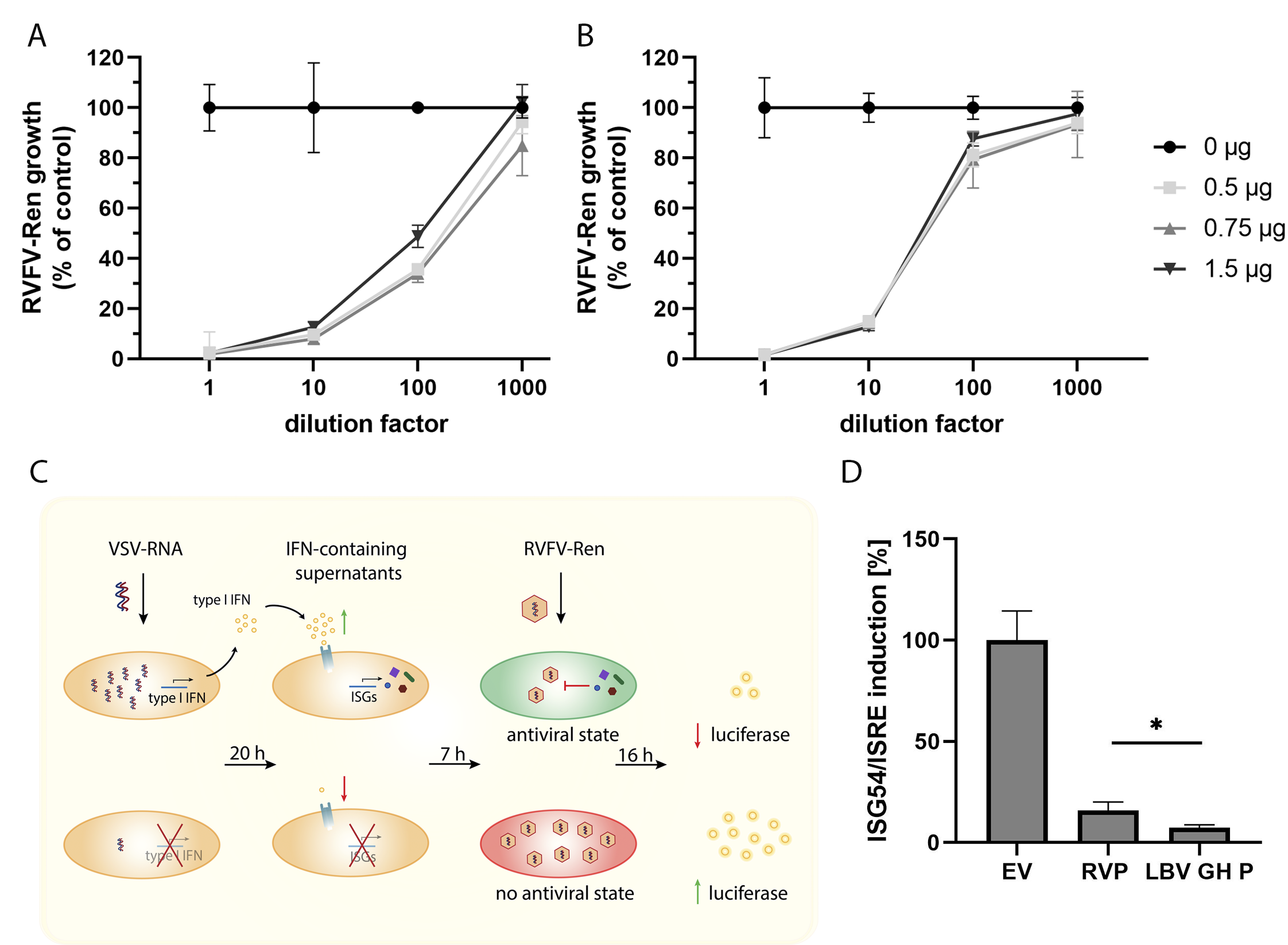

Supplement: S1 Fig — The functionality of IFN signaling pathways was analyzed in A549 (A) and EidLu/20.2 (B) cells. After seeding cells, increasing amounts of total RNA from Vesicular stomatitis virus (VSV)-infected cells were transfected as indicated. After 20 h the supernatants were collected, diluted as indicated, and added to naïve cells of the respective cell line. After 7 h cells were infected with RVFV-Ren (MOI 0.002), incubated for 16 h, lysed and luciferase signal was measured. Virus growth was calculated relative to the mock-treated cells (0 μg) for the respective dilution. High values indicate increased virus replication and low amounts of bioactive IFN in the supernatants. Experiments were performed in triplicate and mean values and SD are shown. The experimental procedure is depicted in (C). Upon detection of VSV-RNA type I IFNs are produced and secreted. Incubation of cells with IFN-containing supernatants induces an antiviral state characterized by the transcription of interferon-stimulated genes (ISGs). RVFV-Ren reporter virus growth is inhibited by various ISG proteins in IFN stimulated cells. EidLu/20.2 cells were transfected with RL and FF luciferase reporter plasmids (pISG54-luc) and 50 ng of pCAGGS empty vector (EV) or pCAGGS vector encoding Rabies virus P protein (RVP) or LBV-GH P protein (LBV GH P). Cells were stimulated by recombinant IFN treatment (100 U/ml) and lysed after 18 h. Luciferase signal was measured and ISG54/ISRE induction relative to EV was calculated (D). All experiments were performed in triplicate and mean values and SD were calculated. * = p<0.05; determined by student’s t-test (see S2 Table for details). (TIF) [file pone.0264450.s001.tif]

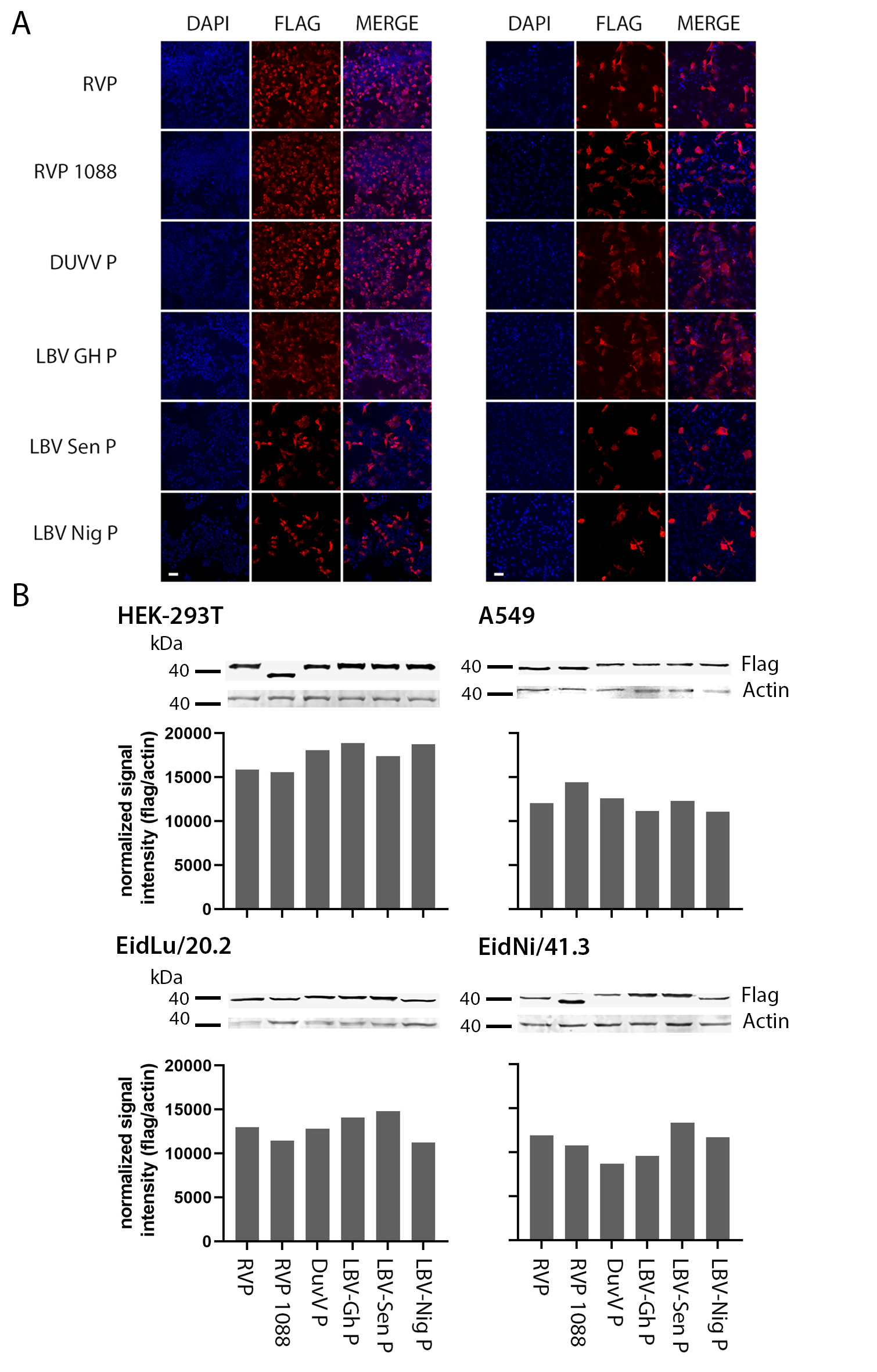

Supplement: S2 Fig — Human kidney (HEK-293T) and E. helvum lung (EidLu/20.2) cells were transfected with pCAGGS vector encoding P proteins from human- and bat-associated lyssaviruses (Rabies virus P protein (RVP); RVP street rabies strain 1088 (RVP 1088); Duvenhage virus P protein (DUVV P); LBV Ghana isolate P protein (LBV-GH P); LBV Senegal isolate P protein (LBV-Sen P); LBV Nigeria isolate P protein (LBV-Nig P)). (A) For immunofluorescence assay cells were seeded on glass coverslips in 24-well plates and transfected with 0.5 μg plasmid DNA per well. Cell fixation and lysis were performed 24 h after transfection. For immunofluorescence microscopy, cells were stained with mouse-anti-FLAG antibody (1:100) and goat anti-mouse-Cy3 antibody (1:200). Cellular DNA was stained with DAPI. Bars represent 50 μm. (B) For Western blot analysis, cell lysates from transfected (2 μg/6-well, respectively) HEK-293T, and A549, EidLu/20.2, and EidNi/41.3 were prepared 48 h post-transfection with Pierce RIPA lysis buffer (Thermo Fisher). Protein lysates and Spectra™ Multicolor Broad Range Protein Ladder (Thermo Fisher) were separated by SDS-PAGE using 10% polyacrylamide gels (120 V, 45 min). Western blot analysis was performed with mouse-anti-FLAG (1:3,000; Sigma-Aldrich) and mouse-anti-β-actin (1:5,000; Sigma-Aldrich) primary antibodies and fluorescently-labeled goat-anti-mouse (1∶15,000; 800 nm; Licor), or goat-anti-rabbit (1:15,000; 680 nm; Licor) secondary antibody. Fluorescent signal was visualized with the Licor Odyssey CLx imaging system (Licor), fluorescent signal intensities were measured and FLAG-based signal intensity was normalized to actin reference protein using the Image Studio™ software package. (TIF) [file pone.0264450.s002.tif]
