# Supplementary material for: Reduced IFN-ß inhibitory activity of Lagos bat virus phosphoproteins in human compared to Eidolon helvum bat cells
Source: PLoS One. 2022 Mar 8;17(3):e0264450. doi: 10.1371/journal.pone.0264450 (PMC8903296; doi:10.1371/journal.pone.0264450)
Supplement: S1 Table — (DOCX) [file pone.0264450.s003.docx]

**S1 Table: PCR primers and probes**

| Name | Sequence | Source |
| --- | --- | --- |
| LBV_f | TGCCAAATTAGACCCAGATGATGT | In-house |
| LBV_p | CTTATCTGGCCGCAGCAATGCAGTTC |  |
| LBV_r | CCTCAGGACACACTCCTTCGA |  |
| bACTB_for | GGCTCCCAGCACAATGAAGA | (1) |
| bACTB_rev | GGAGCCGCCGATCCA |  |
| bACTB_p | FAM-CAAGATCATTGCGCCCCCTGAGC-IBFQ |  |
| hACTB_for | GAGGCATCCTCACCCTGAAG | (1) |
| hACTB_rev | ACCAACTGGGACGACATGGA |  |
| hACTB_p | FAM-CCCCATCGAGCACGGCATCG-IBFQ |  |
| hTBP_f | GCTGCGGTAATCATGAGGATAAG | (1) |
| hTBP_rev | TGCACACCATTTTCCCAGAA |  |
| hTBP_p | FAM-AGCCACGAACCACGGCACTGATTTT IBFQ |  |
| hCCL5_f | TGCCCACATCAAGGAGTATTTC | In-house |
| hCCL5_rev | CCATCCTAGCTCATCTCCAAAG |  |
| hCCL5_p | FAM-TCACCCGAAAGAACCGCCAAGT IBFQ |  |
| hIFIT1_f | CCTGGAGTACTATGAGCGGGC | (2) |
| hIFIT1_rev | TGGGTGCCTAAGGACCTTGTC |  |
| hIFIT1_p | FAM-ACAGAGTTCTCAAAGTCAGCAGCCAGTCTCAGG IBFQ |  |
| hMX1_f | TTCAGCACCTGATGGCCTATC | (2) |
| hMX1_rev | TGGATGATCAAAGGGATGTGG |  |
| hMX1_p | FAM-CAGGAGGCCAGCAAGCGCCATC IBFQ |  |
| bIFNB1_for: | CAGCTATTTCCATGAGCTACAACTTG | (1) |
| bIFNB1_rev | TTAACTGCCACAGGAGCTTCAG |  |
| bIFNB1_p | FAM-TCGATTCCAACAAAGAAGCAGCAATTTAGC-IBFQ |  |
| hIFNB1_for | GAACTTTGACATCCCTGAGGAGATT | (2) |
| hIFNB1_rev | GGAGCATCTCATAGATGGTCAATG |  |
| hIFNB1_p | FAM-CAGCAGTTCCAGAAGGAGGACGCC-IBFQ |  |
| Eidolon IFNb-P Screen fw | GAATGTGAAGTACAGCATAGGG | In-house |
| Eidolon IFNb-P Screen rev | GCTTCAGGCAGGCTAAATTGC | In-house |
| MyoLucF378 | GTAAGTTGGTACAGCCACTATG | In-house |
| MyoLucR1082 | GGAGCGTGCCATAGTTCATG | In-house |
| bIFNp F1 | gtgccagatgggtactagattttttggggtga | In-house |
| bIFNpF2 | TCATTCCATTGTAATTGAAAAATATAAATGA | In-house |
| bIFNp R3.Afr | CACAACAGGAGAGCAATTTG | In-house |
| p189_KpnI_fw2 | CGGGGTACCAATTGAAAAATATAAATGACAAAAGAAAAC | In-house |
| p189_complete_XhoI_rev2 | ATCTCGAGGAGCCTGGGCTATTTATACGGTCATC | In-house |
| p125_KpnI_fw2 | CGGGGTACCGTTTTAGAAACTACTAAAATGTAAATGAC | In-house |
| p125_complete_XhoI_rev2 | ATCTCGAGGGGTATGGCCTATTTA TATGAGATGGTCC | In-house |
| pGL4.10-luc2_MCS_Screen_fw | GATAGTACTAACATACGCTCTCC | In-house |
| pGL4.10-luc2_MCS_Screen_rev | AGCGCTTCATGGCTTTGTGC | In-house |

1. Biesold SE, Ritz D, Gloza-Rausch F, Wollny R, Drexler JF, Corman VM, et al. Type I interferon reaction to viral infection in interferon-competent, immortalized cell lines from the African fruit bat Eidolon helvum. Plos One. 2011;6(11):e28131.

2. Holzinger D, Jorns C, Stertz S, Boisson-Dupuis S, Thimme R, Weidmann M, et al. Induction of MxA gene expression by influenza A virus requires type I or type III interferon signaling. Journal of Virology. 2007;81(14):7776-85.
