# Supplementary material for: Reduced IFN-ß inhibitory activity of Lagos bat virus phosphoproteins in human compared to Eidolon helvum bat cells
Source: PLoS One. 2022 Mar 8;17(3):e0264450. doi: 10.1371/journal.pone.0264450 (PMC8903296; doi:10.1371/journal.pone.0264450)
Supplement: S2 Table — (DOCX) [file pone.0264450.s004.docx]

**S2 Table: Results of unpaired student’s t-tests**

| Input | P value | Confidence | Figure |
| --- | --- | --- | --- |
| **A549** |  |  |  |
| RVFV vs VSV-RNA | 0.0015 | ** | 1B |
| **EidLu/20.2** |  |  |  |
| RVFV vs VSV-RNA | <0.0001 | **** | 1B |
| **A549 vs. EidLu/20.2** |  |  |  |
| 72 h | 0.0305 | * | 1C |
| 96 h | 0.0017 | ** | 1C |
| **HEK-293T** |  |  |  |
| RVP vs LBV GH P | <0.0001 | **** | 2A |
| **HEK-293T** |  |  |  |
| RVP vs LBV GH P | <0.0001 | **** | 2B |
| **HEK-293T** |  |  |  |
| *CCL5:* EV vs RVP | 0.0034 | ** | 2C |
| *CCL5:* EV vs LBV GH P | 0.0060 | ** | 2C |
| *IFIT1:* EV vs RVP | <0.0001 | **** | 2C |
| *IFIT1:* EV vs LBV GH P | 0.0023 | ** | 2C |
| *MX1:* EV vs RVP | <0.0001 | **** | 2C |
| *MX1:* EV vs LBV GH P | 0.0021 | ** | 2C |
| **HEK-293T** |  |  |  |
| RVP vs RVP 1088 | 0.9076 | ns | 4A |
| RVP vs DUVV P | 0.0652 | ns | 4A |
| RVP vs LBV GH P | <0,0001 | **** | 4A |
| RVP vs LBV Sen P | <0,0001 | **** | 4A |
| RVP vs LBV Nig P | <0,0001 | **** | 4A |
| **A549** |  |  |  |
| RVP vs RVP 1088 | 0.0011 | ** | 4B |
| RVP vs DUVV P | 0.4931 | ns | 4B |
| RVP vs LBV GH P | 0.0039 | ** | 4B |
| RVP vs LBV Sen P | 0.0134 | * | 4B |
| RVP vs LBV Nig P | 0.0247 | * | 4B |
| **EidLu/20.2** |  |  |  |
| RVP vs RVP 1088 | 0.2137 | ns | 4C |
| RVP vs DUVV P | 0.6782 | ns | 4C |
| RVP vs LBV GH P | 0.4337 | ns | 4C |
| RVP vs LBV Sen P | 0.3008 | ns | 4C |
| RVP vs LBV Nig P | 0.1401 | ns | 4C |
| **EidNi/41.3** |  |  |  |
| RVP vs RVP 1088 | 0.0054 | ** | 4D |
| RVP vs DUVV P | 0.0068 | ** | 4D |
| RVP vs LBV GH P | 0.0284 | * | 4D |
| RVP vs LBV Sen P | 0.0023 | ** | 4D |
| RVP vs LBV Nig P | 0.0108 | * | 4D |
| **EidLu/20.2** |  |  |  |
| RVP vs LBV GH P | 0.0258 | * | S1 Fig D |
