# Supplementary material for: Reduced IFN-ß inhibitory activity of Lagos bat virus phosphoproteins in human compared to Eidolon helvum bat cells
Source: PLoS One. 2022 Mar 8;17(3):e0264450. doi: 10.1371/journal.pone.0264450 (PMC8903296; doi:10.1371/journal.pone.0264450)

Figure S2

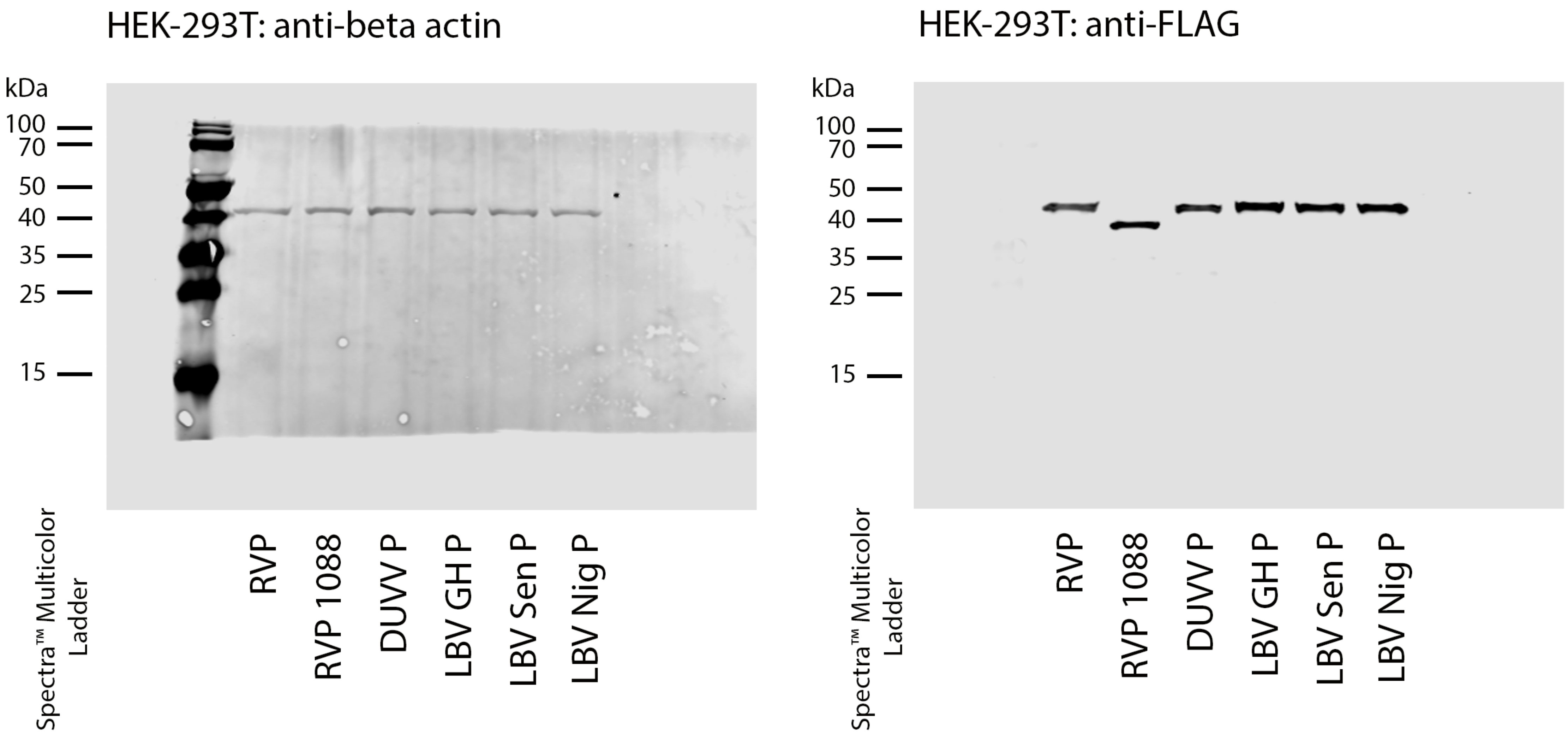

HEK-293T: anti-beta actin
HEK-293T: anti-FLAG
kDa
100
70
50
40
35
25
15
Spectra™ Multicolor Ladder
RVP
RVP 1088
DUVV P
LBV GH P
LBV Sen P
LBV Nig P

Figure S2

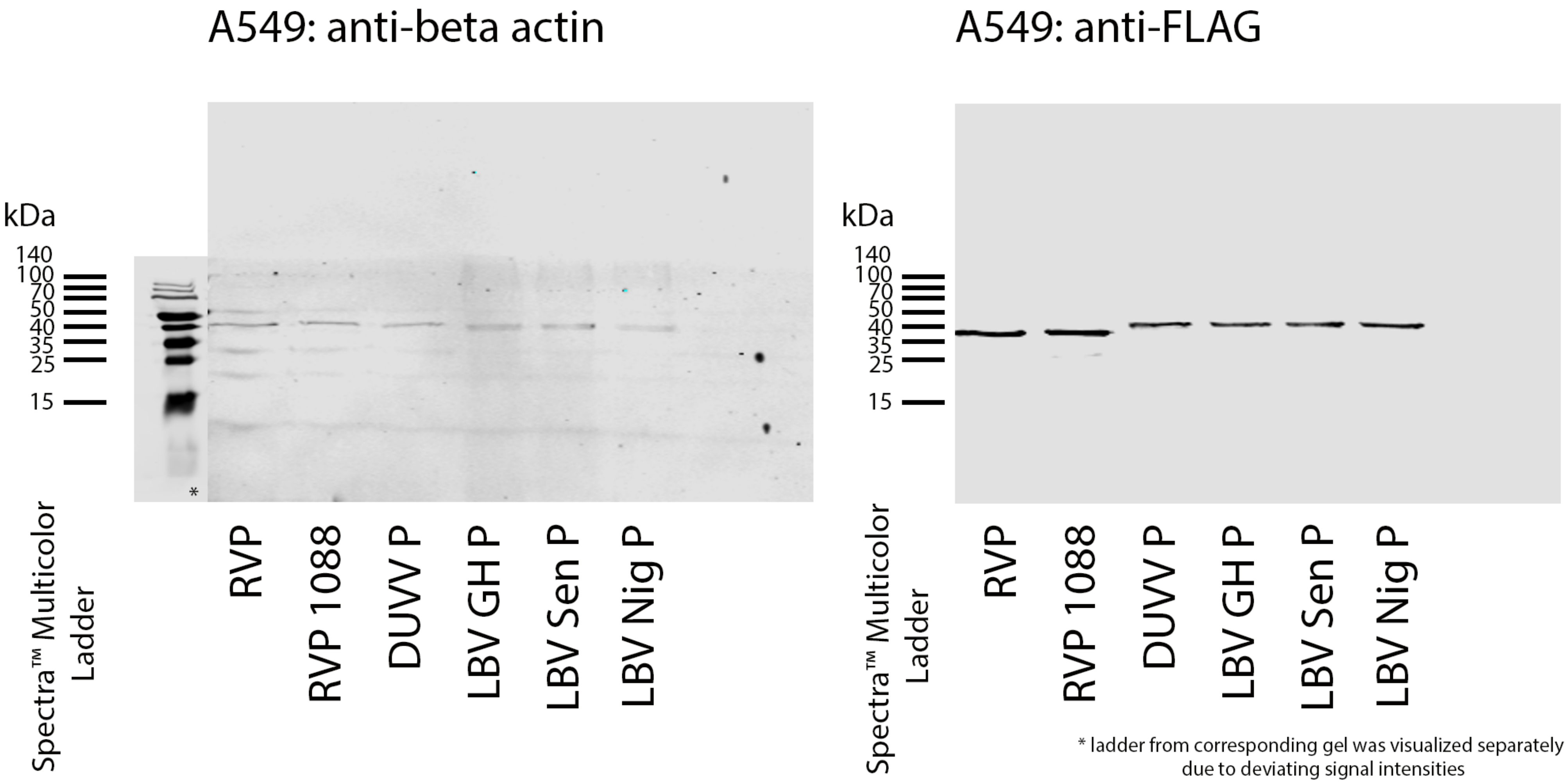

* ladder from corresponding gel was visualized separately due to deviating signal intensities

Figure S2

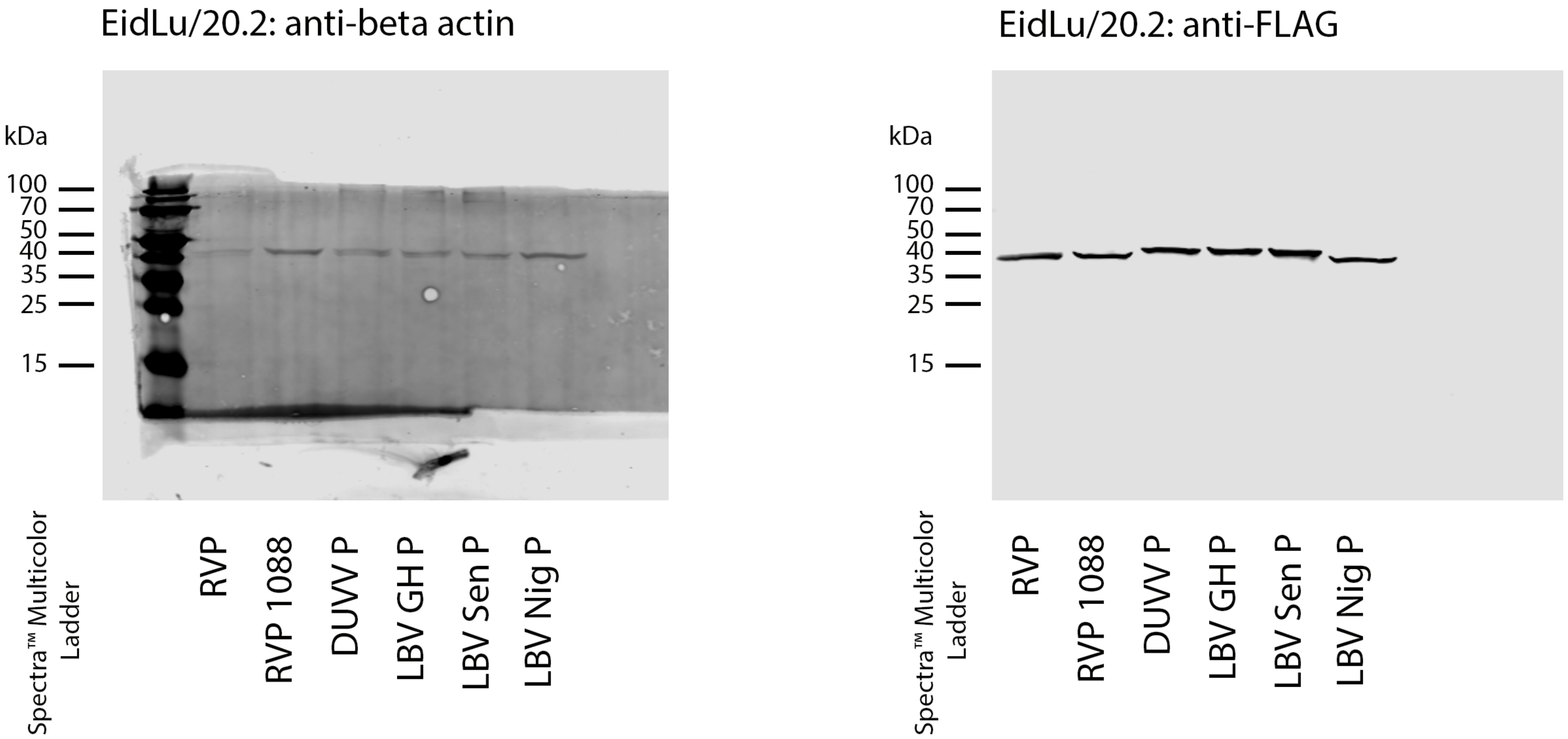
EidLu/20.2: anti-beta actin
EidLu/20.2: anti-FLAG
kDa
100
70
50
40
35
25
15
Spectra™ Multicolor Ladder
RVP
RVP 1088
DUVV P
LBV GH P
LBV Sen P
LBV Nig P

Figure S2

EidNi/41.3: anti-beta actin

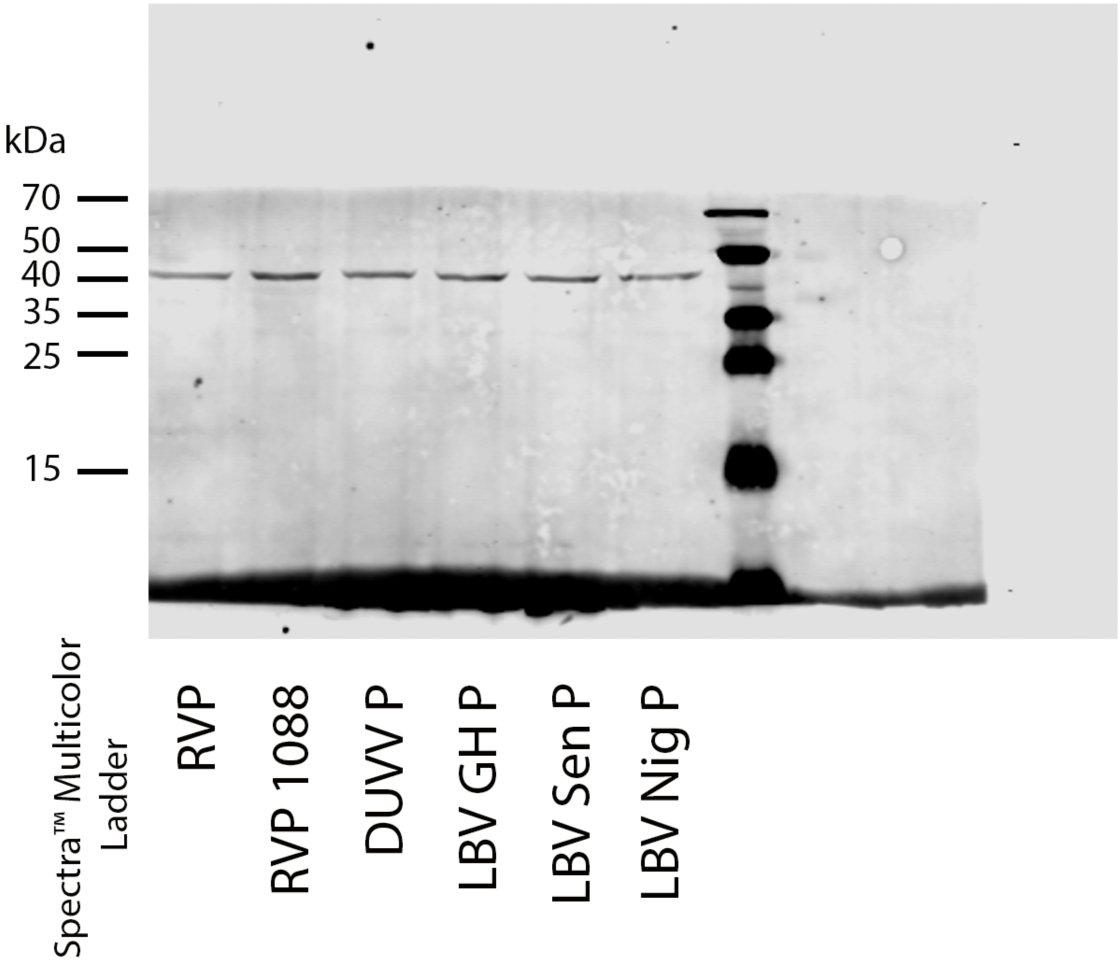

EidNi/41.3: anti-FLAG

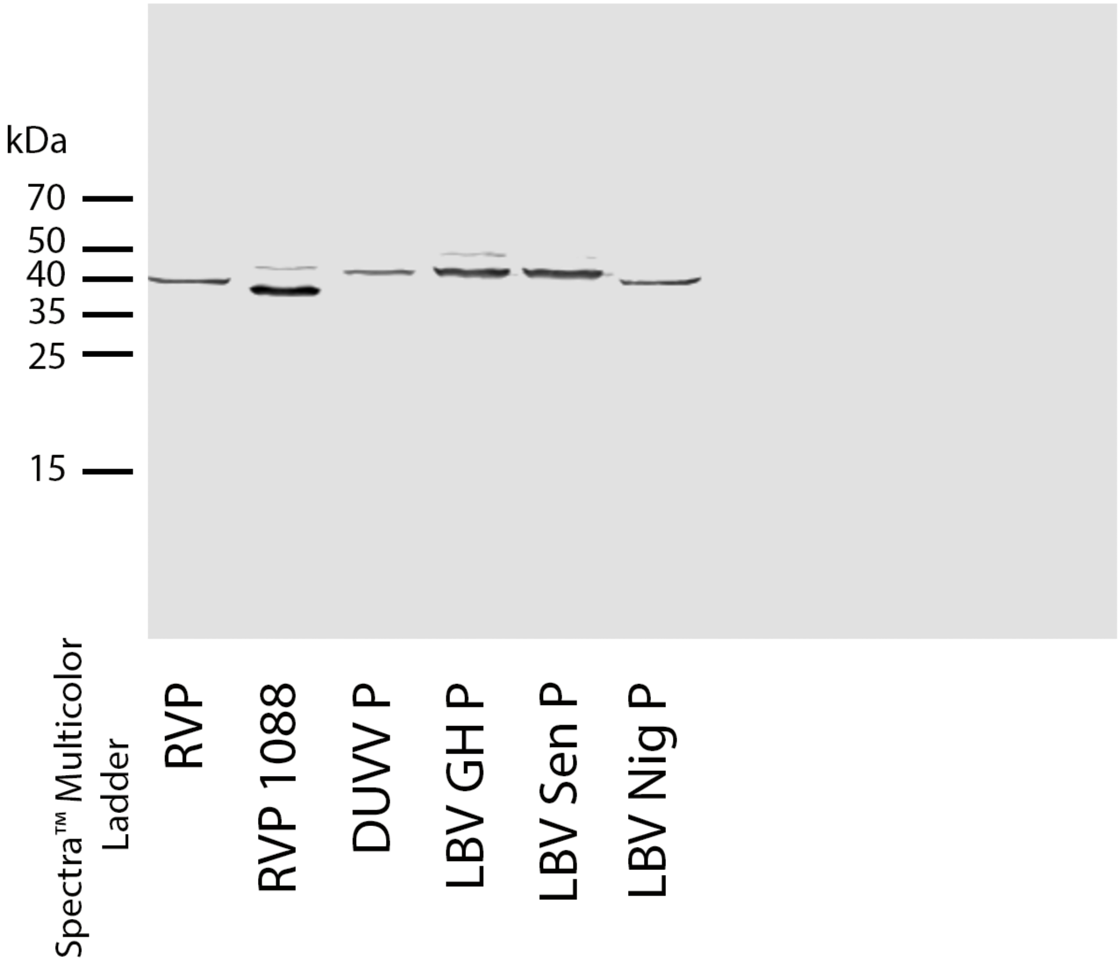

Supplement: S1 File — (PDF) [file pone.0264450.s005.pdf]
